# Neurodevelopmental risk copy number variants in adults with intellectual disabilities and comorbid psychiatric disorders

**DOI:** 10.1192/bjp.2017.65

**Published:** 2018-05

**Authors:** Johan H. Thygesen, Kate Wolfe, Andrew McQuillin, Marina Viñas-Jornet, Neus Baena, Nathalie Brison, Greet D'Haenens, Susanna Esteba-Castillo, Elisabeth Gabau, Núria Ribas-Vidal, Anna Ruiz, Joris Vermeesch, Eddy Weyts, Ramon Novell, Griet Van Buggenhout, André Strydom, Nick Bass, Miriam Guitart, Annick Vogels

**Affiliations:** 1 Division of Psychiatry, University College London, London, UK; 2 Genetics Laboratory, UDIAT-Centre Diagnostic, Hospital de Sabadell, Parc Taulí, Hospital Universitari, Institut d'Investigació i Innovació Parc Taulí I3PT, Universitat Autònoma de Barcelona, Sabadell, Spain; 3 Department of Human Genetics, Centre for Human Genetics, University Hospitals Leuven, Leuven, Belgium; 4 St Camillus Psychiatric Hospital, Bierbeek, Belgium; 5 Mental Health and Intellectual Disability Specialized Service, Institut Assistència Sanitària (IAS), Parc Hospitalari Martí i Julià, Girona, Spain; 6 Genetics Laboratory, UDIAT-Centre Diagnostic, Hospital de Sabadell, Parc Taulí, Hospital Universitari, Institut d'Investigació i Innovació Parc Taulí I3PT, Universitat Autònoma de Barcelona, Sabadell, Spain; 7 Mental Health and Intellectual Disability Specialized Service, Institut Assistència Sanitària (IAS), Parc Hospitalari Martí i Julià, Girona, Spain; 8 Genetics Laboratory, UDIAT-Centre Diagnostic, Hospital de Sabadell, Parc Taulí, Hospital Universitari, Institut d'Investigació i Innovació Parc Taulí I3PT, Universitat Autònoma de Barcelona, Sabadell, Spain; 9 Department of Human Genetics, Centre for Human Genetics, University Hospitals Leuven, Leuven, Belgium; 10 St Camillus Psychiatric Hospital, Bierbeek, Belgium; 11 Mental Health and Intellectual Disability Specialized Service, Institut Assistència Sanitària (IAS), Parc Hospitalari Martí i Julià, Girona, Spain; 12 Department of Human Genetics, Centre for Human Genetics, University Hospitals Leuven, Leuven, Belgium; 13 Division of Psychiatry, University College London and Department of Forensic and Neurodevelopmental Science, Institute of Psychiatry, Psychology and Neuroscience, Kings College London, London, UK; 14 Division of Psychiatry, University College London, London, UK; 15 Genetics Laboratory, UDIAT-Centre Diagnostic, Hospital de Sabadell, Parc Taulí, Hospital Universitari, Institut d'Investigació i Innovació Parc Taulí I3PT, Universitat Autònoma de Barcelona, Sabadell, Spain; 16 Department of Human Genetics, Centre for Human Genetics, University Hospitals Leuven, Leuven, Belgium

## Abstract

**Background:**

Copy number variants (CNVs) are established risk factors for neurodevelopmental disorders. To date the study of CNVs in psychiatric illness has focused on single disorder populations. The role of CNVs in individuals with intellectual disabilities and psychiatric comorbidities are less well characterised.

**Aims:**

To determine the type and frequency of CNVs in adults with intellectual disabilities and comorbid psychiatric disorders.

**Method:**

A chromosomal microarray analysis of 599 adults recruited from intellectual disabilities psychiatry services at three European sites.

**Results:**

The yield of pathogenic CNVs was high – 13%. Focusing on established neurodevelopmental disorder risk loci we find a significantly higher frequency in individuals with intellectual disabilities and comorbid psychiatric disorder (10%) compared with healthy controls (1.2%, *P*<0.0001), schizophrenia (3.1%, *P*<0.0001) and intellectual disability/autism spectrum disorder (6.5%, *P* < 0.00084) populations.

**Conclusions:**

In the largest sample of adults with intellectual disabilities and comorbid psychiatric disorders to date, we find a high rate of pathogenic CNVs. This has clinical implications for the use of genetic investigations in intellectual disability psychiatry.

**Declaration of interest:**

None.

Neurodevelopmental disorders are a group of disorders that are characterised by perturbed neurodevelopment –intellectual disabilities, autism spectrum disorder (ASD) and schizophrenia are all considered to be neurodevelopmental disorders.^1^ A proportion of the risk for neurodevelopmental disorders can be attributed to a class of genetic variants known as copy number variants (CNVs).^2^ A CNV is typically defined as a segment of DNA >1 kilobase, which is present at a higher (duplication) or lower (deletion) copy number compared with a reference genome.^3^ Intellectual disability has its onset in childhood and initially manifests with failure to meet developmental milestones, known as developmental delay. In adulthood, a clinical diagnosis of intellectual disability is typically given when there are both deficits in adaptive and intellectual functioning (IQ score <70). Intellectual disability can occur in isolation or in combination with a range of somatic, psychiatric and behavioural disorders. Association studies have shown the involvement of CNVs in psychiatric risk, in particular CNVs have been strongly implicated in the aetiology of schizophrenia^4^ and ASD.^5^ Furthermore, investigations in large paediatric cohorts have revealed CNV regions that are significantly associated with intellectual disabilities.^6^ Many of these CNVs operate across traditional diagnostic boundaries: for example, 11 of the CNVs associated with intellectual disabilities are also risk factors for schizophrenia.^7^ The neurodevelopmental disorder risk CNVs that have been identified to date confer moderate to large risk (odds ratio 1.5 to ≥50),^7^ and therefore have important clinical implications for affected individuals and at-risk family members.

A major challenge in the clinical interpretation of CNVs is the variable penetrance and expressivity of many neurodevelopmental disorder risk CNVs. For example, not all individuals with a particular CNV display a neurodevelopmental phenotype (penetrance) and not all individuals express a severe phenotype (expressivity).^8^ A large proportion, approximately 50%, of adult intellectual disability is idiopathic or of unknown cause.^9^ Chromosomal microarray analysis (CMA), the group of tests used to detect CNVs, have been one of the recommended first-tier tests for clinical investigation of idiopathic intellectual disabilities since around 2010 and have primarily been undertaken in paediatric populations.^10^ Testing of adults with intellectual disabilities is particularly important for elucidating the relationship between rare CNVs and late-onset medical and psychiatric phenotypes. Indeed, the highest burden of pathogenic CNVs may be present in adults expressing comorbid neurodevelopmental phenotypes.

## Method

To the best of our knowledge, this study is the first multipopulation analysis of CNVs in adults with intellectual disabilities and psychiatric comorbidities and represents the largest sample of its kind to date. We aimed to determine: (a) the frequency of known neurodevelopmental disorder risk CNVs compared with large population cohorts from the literature (healthy controls, individuals with intellectual disabilities/ASD and schizophrenia);^7^ (b) the overall rate of pathogenic CNVs; (c) the relationship between pathogenic CNVs, level of intellectual disability and comorbid psychiatric diagnoses; and (d) likely pathogenic CNVs affecting neurodevelopmental candidate genes.

### Participant recruitment

The GENMID (GENetics of Mental disorders in Intellectual Disability) consortium is comprised of three primary research groups based in Catalonia, Spain; Leuven, Belgium; and England, UK. In Catalonia participants were identified between 2009 and 2012 from the mental health intellectual disabilities regional community service at Parc Hospitalari Martí i Julià, Girona. In Leuven, participants were recruited between 2005 and 2015 at the regional in-patient psychiatric unit for adults with intellectual disabilities at the St Camillus Psychiatric Hospital, Bierbeek. Initially, only patients diagnosed with psychosis were recruited, but recruitment was later extended to other psychiatric phenotypes. In England, participants were recruited by consultant psychiatrists in intellectual disabilities between 2012 and 2015 from intellectual disability psychiatry case-loads at 32 National Health Service (NHS) trusts and 1 non-NHS provider.

Written informed consent was obtained for all participants with capacity to consent and consultee/guardian advice was sought in the absence of this. Approval in England was granted by the North Wales Research Ethics Committee West, reference 11/WA/0370. In Catalonia approval was granted by Catalonia Corporació Sanitària Parc Taulí Ethics Coomittee reference 2009/582. In Leuven approval was granted by the Commissie Medische Ethiek van de Universitaire Ziekenhuizen KU Leuven, reference S54583 (ML8614).

### Recruitment criteria

All sites recruited adults over the age of 18 years. Participants had idiopathic intellectual disabilities, defined as no clear genetic or environmental cause of intellectual disabilities as detailed in their medical records. Participants had one or more comorbid psychiatric diagnoses and/or significant challenging behaviours.

### Phenotypic assessments

For all sites the intellectual disability levels are in accordance with the ICD-10 ranges (<20 profound intellectual disabilities, 20–34 severe intellectual disabilities, 35–49 moderate intellectual disabilities, 50–69 mild intellectual disabilities, 70–84 borderline intellectual disabilities).^11^ For further analyses, the <20–49 ranges were collapsed into a severe category and the 50–84 ranges were collapsed into a mild category. All sites identified psychiatric diagnoses from medical records and/or informants. Psychiatric diagnoses were converted from DSM-IV^12^ to ICD-10 criteria (with agreement between two psychiatrists).

### Genetic analysis and CNV calling

DNA was extracted from blood and saliva samples. Samples from Catalonia were analysed using the 400 K Agilent platform (Agilent Technologies, Santa Clara, California, USA) at the Genetics Laboratory, UDIAT-Centre Diagnòstic, Parc Taulí Hospital Universitari. Samples from Leuven were analysed on the CytoSure ISCA oligoarray set (OGT, Oxford, UK) at the Constitutional Cytogenetics Unit of the Center of Human Genetics. Samples from England were analysed on the NimbleGen 135 K platform (87%) (Roche NimbleGen, Madison, Wisconsin, USA) and the Cytoscan 750 K platform (13%) (Affymetrics, Santa Clara, California, USA) at the North East Thames Regional Genetics Service Laboratory. CNV calling took place at the respective clinical laboratories, in keeping with internal laboratory protocols based on the American College of Medical Genetics best guidelines^1^,^3^ or the Association of Clinical Genetic Science Best Practice Guidelines.^1^,^4^

CNVs reported by the clinical laboratories were classified into three categories: pathogenic, uncertain clinical significance and benign. The genome coordinates for all sites are reported according to the National Center for Biotechnology Information (NCBI) human genome build 37 (hg19, February 2009). All pathogenic CNVs were fed back to the participants' treating psychiatrist.

### Analysis methodology

We aimed to compare the rate of known rare neurodevelopmental disorder risk CNVs in our cohort with rates in other patient populations. We used a list of 63 neurodevelopmental disorder risk CNVs from Rees *et al*,^7^ originally derived from Coe *et al.*^6^ The patient population rates in healthy controls, ID/ASD (the name given by Rees *et al* to a severe developmental disorders cohort), and schizophrenia were derived from Rees *et al*,^7^ where further information can be found about the respective samples. Neurodevelopmental disorder CNV carriers were identified using the criteria outlined in Kendall *et al*,^1^,^5^ also used by Rees *et al*^7^ (George Kirov, personal communication via email, 27/10/2016) (see Supplementary Table 1 available at https://doi.org/10.1192/bjp.2017.65). CNVs fulfilling these calling criteria were classified as pathogenic and are included in the diagnostic yield. Duplications (dup) or deletions (del) of the same chromosomal region were counted as separate loci (for example 22q11.2 del and dup). A rate percentage was calculated to enable comparisons between different sample sizes and chi-squared tests were used to determine the population differences. The significance level has been adjusted to *P* = 0.01 to account for multiple pairwise comparisons.

To determine the CMA yield each individual was grouped by the most pathogenic CNV detected. Between site discrepancies were reclassified in accordance with Kearney *et al*^1^,^3^ (see Supplementary Table 2). CNVs designated as of uncertain clinical significance were reclassified into likely benign or likely pathogenic using this methodology. We examined all likely pathogenic CNVs for recurrence and describe the main loci that have been implicated as neurodevelopmental disorder risk factors in the current literature.

Finally, we performed chi-squared tests (or Fisher's exact where there were five or less individuals) to examine the differences between psychiatric diagnoses, intellectual disability level and CNV pathogenicity. Since many of the comorbid diagnoses are correlated and thus are non-independent, correction of *P*-values through Bonferroni or other methods was deemed too stringent. Thus, we present all *P*-values uncorrected for multiple testing as recommended by several authors,^1^,^6^^,^^1^,^7^ while indicating the number of tests performed if all comparisons are not presented. All analyses were performed using R version 3.3.1.^1^,^8^

## Results

We recruited 599 adults (Catalonia, *n* = 80; Leuven, *n* = 272; and England, *n* = 247) with intellectual disabilities and one or more comorbid psychiatric diagnoses/challenging behaviours (376 (62.8%) male, mean age 43.2). Just more than half of the sample (50.8%) had severe intellectual disabilities and the remainder had mild intellectual disabilities. Each participant had on average 1.6 comorbid psychiatric diagnoses, with pervasive developmental disorders being the most frequent diagnosis (25%), followed by unspecified non-organic psychosis (20%) (Table 1 and Supplementary Table 3). The average number of CNVs per participant was 12.5 (7.4 deletions and 5.5 duplications). Analysis of mean CNV size revealed that pathogenic CNVs were the largest followed by likely pathogenic, and both categories were significantly larger than likely benign and benign CNVs (Supplementary Fig. 1). In line with guidelines of CNV categorisation our results follow the expected size distribution in that pathogenic CNVs are the largest.

**Table 1:** Descriptive summary of GENetics of Mental disorders in Intellectual Disability (GENMID) cohort

	GENMID
Demographics	
Participants, *n*	599
Ratio (male/female)	1.7 (376/223)
Age, mean (s.d.)	43.2 (14.1)
Intellectual disabilities level, %	
Mild	49.2
Severe	50.8
Psychiatric diagnoses	
Number of comorbid diagnoses, mean (range)	1.6 (1–5)
F84 Pervasive developmental disorders, *n* (%)	148 (25)
F29 Unspecified non-organic psychosis, *n* (%)	121 (20)
F61 Mixed and other personality disorders, *n* (%)	108 (18)
Challenging behaviours, *n* (%)	95 (16)
F32 Depressive episode, *n* (%)	86 (14)
F41 Other anxiety disorders, *n* (%)	60 (10)
F20 Schizophrenia, *n* (%)	49 (8)
F31 Bipolar affective disorder, *n* (%)	47 (8)
F90 Hyperkinetic disorders, *n* (%)	41 (7)
F42 Obsessive–compulsive disorder, *n* (%)	37 (6)
F43 Reaction to severe stress and adjustment disorders, *n* (%)	27 (5)
F39 Unspecified mood disorder, *n* (%)	25 (4)

### Neurodevelopmental CNV frequency analysis

In our sample, we found CNVs in 23 of the 63 neurodevelopmental disorder loci described by Rees *et al*.^7^ At these 23 loci we identified 58 CNV carriers, with 2 individuals carrying two risk CNVs. The rate percentage in our sample (rate of participants with a neurodevelopmental disorder CNV) is 10.0%, whereas the rate percentage, determined from the data presented in Rees *et al*, is 6.5% in the intellectual disability/ASD, 3.1% in the schizophrenia and 1.2% in the healthy control populations (Table 2).^7^ The neurodevelopmental disorder loci frequencies are most comparable with the intellectual disability/ASD population, a sample that consisted mainly of children with developmental delay/intellectual disabilities and/or ASD.^6^^,^^7^ However, we still observe significantly higher proportions of neurodevelopmental disorder CNVs in our intellectual disabilities and comorbid psychiatric diagnosis sample, 3.5% higher (95% CI 1–6, *P* = 0.00084).

**Table 2:** Rate (%) of copy number variant frequencies^a^ at 63 neurodevelopmental disorder risk loci in the GENetics of Mental disorders in Intellectual Disability (GENMID) cohort compared with populations rates reported by Rees *et al* (2016)^7^

Sample	Sample size	Rate of the 63 neurodevelopmental disorder loci, %	Rate difference, % (95% CI),	*P*
Healthy control	26 628	1.2	8.8 (6.3–11)	2.8 × 10^–72^
Schizophrenia	20 403	3.1	7 (4.5–9.5)	9.7 × 10^–21^
Intellectual disability/autism spectrum disorder	29 085	6.5	3.5 (1–6)	8.4 × 10^–4^
GENMID	599	10.0	–	–

a. Rate percentage differences, 95% CI and *P*-values for rate comparisons are indicated.

**Fig. 1:**
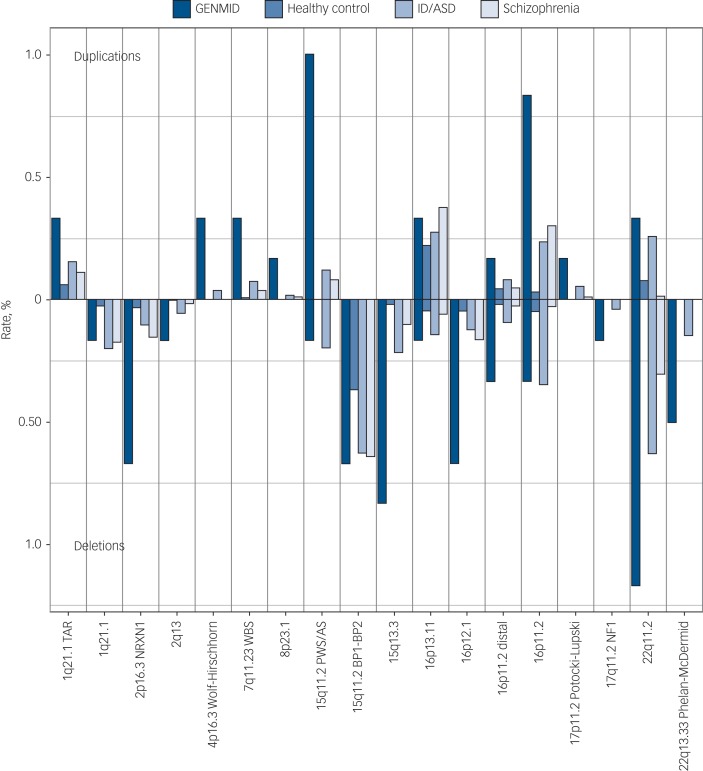
Neurodevelopmental disorder copy number variant frequencies in the GENetics of Mental disorders in Intellectual Disability (GENMID) sample compared with frequencies in healthy controls (*n* = 26 628), intellectual disability/autism spectrum disorder (ID/ASD) (*n* = 29 085) and schizophrenia (*n* = 20 403) cohorts as reported by Rees *et al*.^7^ Rates for deletions extend down from the central line and duplications extend upwards.

The frequencies of the 23 neurodevelopmental disorder CNVs identified in this cohort are shown in Fig. 1. The carrier frequency at each loci was the highest in our sample of people with intellectual disabilities and comorbid mental illness, with the exception of four loci for which we see comparable frequencies with the intellectual disability/ASD cohort. The five most frequent neurodevelopmental disorder CNVs in the GENMID cohort, in order of frequency, are: 22q11.2 del (*n* = 7, 1.2%), 15q11.2 Prader–Willi syndrome/Angelman syndrome (PWS/AS) dup (*n* = 6, 1%), 16p11.2 dup (*n* = 5, 0.8%), 15q13.3 del (*n* = 5, 0.8%) and 16p12.1 del (*n* = 4, 0.7%). A description of all CNV loci and the carrier phenotypes can be found in Supplementary Table 4.

### Pathogenic CNV yield

In the GENMID sample 78 participants (13.0%, 95% CI 10.5–16.0) had at least one pathogenic CNV, with similar yields found at all research sites (Catalonia: 13.8%, Leuven: 14.0%, and England: 11.7%). Pathogenic CNVs comprised those identified at the neurodevelopmental disorder loci previously described and a further 25 CNVs reported as pathogenic by the clinical laboratory services (see Supplementary Table 5). The pathogenic CNVs were predominantly deletions (59.5%). We previously reported a rate of 11% pathogenic CNVs in a subset of 202 of the 247 participants from the England sample.^1^,^9^ When these 202 individuals are removed from the combined sample the diagnostic yield is 13.9%, thus replicating the initial finding.

### Intellectual disability level, psychiatric diagnoses and CNV pathogenicity

We examined group differences between CNV pathogenicity, psychiatric diagnoses and level of intellectual disability. We observe some differences in the proportions of intellectual disabilities and psychiatric diagnoses between the CNV pathogenicity groups (pathogenic, likely pathogenic, likely benign and benign; Supplementary Fig. 2). However, no simple unidirectional relationships were observed. Equally, minor differences in the severity of intellectual disabilities were found between CNV pathogenicity groups, but no overall unidirectional relationship was observed.

### Likely pathogenic CNVs

**Fig. 2:**
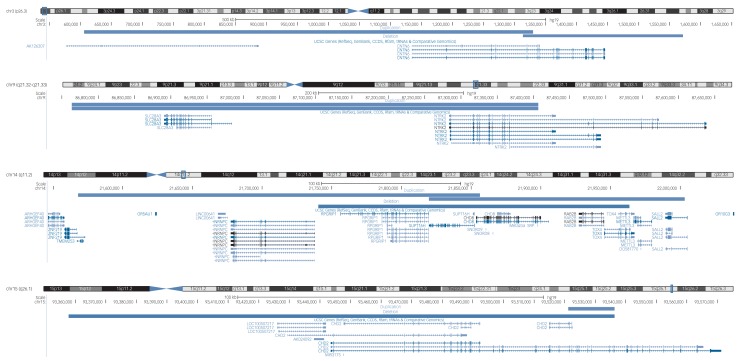
The locations of four overlapping likely pathogenic copy number variants in the GENetics of Mental disorders in Intellectual Disability cohort that are implicated in neurodevelopmental disorders in the literature (UCSC genome browser).

The yield of likely pathogenic CNVs in the sample was 21.5% (95% CI 18.4–25.1). Investigation of recurrent likely pathogenic CNVs revealed 34 CNVs in 16 regions (Supplementary Table 6). Four recurrent CNVs identified here corroborate existing evidence for the involvement of these regions in neurodevelopmental risk (Fig. 2).

First, we identified two carriers of exonic duplications in the *CNTN6* gene at 3p26.3. The CNTN proteins belong to an immunoglobulin super family of cell adhesion molecules and have an important role in neurodevelopmental processes.^20^ *CNTN6* duplications were first identified in patients with ASD^2^,^1^^,^^22^ and later in a patient with intellectual disabilities and facial dysmorphisms.^2^,^3^ A review of 14 patients with *CNTN6* CNVs revealed that both CNV deletions and duplications affecting *CNTN6* are thought to be involved in variable neuropsychiatric phenotypes.^2^,^4^ The participants identified in our study both presented with mild intellectual disabilities. One had schizophrenia and personality disorder, and one had challenging behaviours and had been convicted of a serious criminal offence. Interestingly, the participant with schizophrenia and personality disorder also had a duplication in the *CNTN4* gene. CNVs affecting *CNTN4* are also thought to confer risk for various neurodevelopmental disorders.^2^,^5^

Second, we identified two participants with CNV duplications at the 9q21.32q21.33 locus encompassing the *SLC28A3* and *NTRK2* genes. SLC28A3 is a nucleoside transporter involved in the regulation of multiple processes, including neurotransmission; however, there are no prior reports of its role in psychiatric risk. NTRK2 is a receptor tyrosine kinase with numerous neurodevelopmental functions, including synapse formation and plasticity. Altered *NTRK2* expression has been identified in the brains of patients with schizophrenia.^2^,^6^ One participant had severe intellectual disabilities and bipolar disorder, and the other had mild intellectual disabilities with unspecified non-organic psychosis.

Third, we identified five participants with exonic CNVs in the *CHD* gene family. The CHD proteins are involved in chromatin structure remodelling and the epigenetic regulation of transcription. Three of the participants had exonic CNVs (2 duplications, 1 deletion) in the *CHD8* gene at 14q11.2, which also encompass *SUPT16H*. The protein encoded by the *SUPT16H* gene is thought to be involved in DNA replication and repair. CNV deletions affecting *CHD8* and *SUPT16H* were initially described in children with developmental delay and dysmorphic features.^2^,^7^ Variants in the *CHD8* gene are thought to confer a phenotypic subtype of ASD, comprising macrocephaly, facial dysmorphologies and gastrointestinal abnormalities.^2^,^8^ Both deletions^2^,^9^ and duplications^30^^,^^31^ affecting *CHD8* and *SUPT16H* have been described with variable neurodevelopmental phenotypes. The two participants with CNV duplications both had severe intellectual disabilities, one was diagnosed with schizophrenia and the other with bipolar disorder. The participant with the CNV deletion also had severe intellectual disabilities and ASD.

Finally, we identified two participants with exonic CNVs in the *CHD2* gene at 15q26.1 (one deletion and one duplication). Several patients have been described with CHD2 deletions; with a common phenotype of intellectual disabilities, epilepsy and aggressive challenging behaviours.^3^,^2^^,^^3^,^3^ To our knowledge, a CNV duplication in *CHD*2 has not previously been described in the literature. The deletion carrier had severe intellectual disabilities and schizoaffective disorder, and the duplication carrier had challenging behaviours and bipolar disorder. Both patients also had an epilepsy phenotype.

## Discussion

### Main findings

There is a paucity of research on CNVs in adults with intellectual disabilities and comorbid psychiatric phenotypes. This poses a challenge for genetic counselling of novel and rare CNVs, as descriptions of later-life phenotypes are largely unavailable. Previous investigations in this patient group identified a diagnostic yield of 11% CNVs classed as clinically relevant.^1^,^9^ In this study, utilising data from three European research sites, we replicate this finding with a higher diagnostic yield of 13.0% pathogenic CNVs in 599 participants (or 13.9% with the previously reported cases removed). Given that CMA is being advocated for use in cohorts with schizophrenia, in which the diagnostic yields are lower (between 2.5 and 5%),^3^,^4^^,^^3^,^5^ adults with intellectual disabilities presenting to psychiatric services appear to be a group to prioritise for CMA.

We found CNV carriers at 23 out of the 63 neurodevelopmental disorder loci. It is unsurprising that we did not find carriers in the remaining 40 loci, as these CNVs are very rare with reported frequencies in intellectual disabilities between 0.01 and 0.26% (mean 0.06%).^7^ Presuming that there is an additive effect of having both intellectual disabilities and a comorbid psychiatric disorder, then we would expect to see an increased frequency of the 63 neurodevelopmental disorder CNVs in our cohort. Indeed, the cumulative frequency was significantly higher, compared with both intellectual disability/ASD populations not selected for psychiatric comorbidity and individuals with schizophrenia.

The phenotypic presentation of the neurodevelopmental disorder CNV carriers is highly variable, both in terms of the level of intellectual disability and the psychiatric diagnoses. This indicates a broader role for genes within these CNV loci in conferring general, as opposed to disorder-specific, psychiatric risk. It is possible that this clinical heterogeneity partly reflects the difficulty of diagnosing psychiatric disorders in individuals with intellectual disabilities. Interestingly, at least one CNV carrier at each of the five most frequent loci has a psychosis phenotype. Of particular interest are the four carriers of the 16p12.1 deletion, which was significantly associated with risk for schizophrenia by Rees *et al*.^7^ Three of the four carriers had a psychosis phenotype, offering further support for this locus as a risk factor for both intellectual disabilities and psychotic disorders.

We determined the diagnostic yield of CMA in our cohort. In addition to the CNVs identified at known neurodevelopmental disorder loci, we identified a further 25 CNVs that were reported as pathogenic by the clinical genetic services. The majority of these were large deletion CNVs (1.7–13.2 Mb), which overlapped CNVs described in single case reports in the existing literature. This group of CNVs are likely to be extremely rare and thus would not be observed at high enough frequencies in existing case–control studies. We were unable to identify any clear relationship between intellectual disability level, comorbid psychiatric diagnoses and CNV pathogenicity level. This may indicate that neurodevelopmental disorder CNVs generally have pleiotropic effects; however, research with larger sample sizes would be required to further investigate this.

We investigated likely pathogenic CNVs of uncertain clinical significance. Following a literature review of likely pathogenic CNVs that recur in our sample, we were able to offer further support for the involvement of particular CNV regions in neurodevelopmental and psychiatric risk. Unlike the pathogenic CNVs, the likely pathogenic CNVs supporting existing neurodevelopmental candidate loci were small (<1 Mb) and affected only a small number of genes. There is a growing body of literature for the role of the *CNTN* and *CHD* gene families in risk for intellectual disability and comorbid psychiatric disorders. Again, the phenotypes associated with CNVs affecting these genes appear to be highly variable. It is important to consider the clinical implications of these CNVs, which were not initially reported as pathogenic by the clinical genetics services.

### Implications

Our findings suggest that if CMA is to be offered more widely to adults with intellectual disabilities presenting with mental disorders many would receive a new genetic diagnosis. Such diagnoses have many implications. They provide, at least, a partial explanation for the person's physical and mental health problems, which in turn may have an effect on illness-related beliefs of patients, their families and healthcare professionals. For some CNVs medical and psychiatric associations across the life course are now well described, with implications for clinical management. For example, the 22q11.2 deletion has recommendations for physical health screening, include cardiac, renal and immunology investigations and psychiatric screening.^3^,^6^ Although clinical recommendations for some pathogenic CNVs are less clear, it is important to note that 70% of pathogenic CNVs we identified were in neurodevelopmental disorder risk loci, for which there are existing scientific literature and/or clinical disorder guides available for families and clinicians.^3^,^7^ Identification of pathogenic CNVs also has broader implications for family members, including cascade testing, provision of recurrence risk information and access to support groups. Given the rapid progress of genomic medicine there is a need for research to address questions of clinical utility and adverse outcomes in order to optimise the process of genetic investigation in intellectual disability psychiatry.

### Limitations

One of the limitations of this study is that there were some differences between the populations recruited at the different sites, for example, participants were recruited from in-patient psychiatric services in Leuven and primarily outpatient services in Catalonia and England. Most individuals lacked inheritance data, which is a valuable aid in categorisation of rare variants and may have led to an underestimate of our yield. Different platforms were utilised to detect the CNVs at the different sites; however, as all the platforms used were high resolution this is unlikely to have major effects. Finally, a true estimate of the association between CNV pathogenicity and neurodevelopmental disorder phenotypes would require much larger case–control samples or epidemiological-based studies.

In conclusion, this is to our knowledge the first large multipopulation study of CNVs in individuals with idiopathic intellectual disabilities with comorbid psychiatric disorders. We detected a 13% rate of undiagnosed pathogenic CNVs. From a research perspective, studying this population revealed the highest rate of CNVs at established neurodevelopmental disorder loci and recurrent likely pathogenic CNVs, both offering unique opportunities for further phenotyping of rare variants. Increased clinical testing and research in this population should be a priority for both clinicians and researchers in the field of psychiatric genetics.

## Supplementary material

For supplementary material accompanying this paper visit https://doi.org/10.1017/bjp.2017.65. click here to view supplementary material
