# Supplementary material for: Neurodevelopmental risk copy number variants in adults with intellectual disabilities and comorbid psychiatric disorders
Source: Br J Psychiatry. 2018 May;212(5):287–94. doi: 10.1192/bjp.2017.65 (PMC7083594; doi:10.1192/bjp.2017.65)
Supplement: Supplementary file 1 [file S0007125017000654sup001.docx]

## Supplementary info: High rates of neurodevelopmental risk CNVs in patients with intellectual disabilities and co-morbid psychiatric disorders

## ID Level

The Catalonia site used the K-BIT-II (Kauffman Brief Intelligence Test-II) and ABS-RC2 (Adaptive Behaviour Scale Residence Community-2) to establish the ID level. Only participants with mild and moderate ID were recruited to the study. In Leuven, the level of ID was determined retrospectively (using a combination of Wechsler Adult Intelligence Scale scores, special education level and residence data obtained from medical records). In England, formal IQ test scores (where available) were used in the first instance, followed by an age-equivalent receptive vocabulary score determined using the British Picture Vocabulary Scale 3 (BPVS3) assessment. This has been shown to correlate with IQ (1). Finally, the level of ID was taken from medical records in the absence of both IQ and BPVS3 test scores.

## Additional phenotyping

The psychiatric diagnoses were translated from Diagnostic and Statistical Manual of Mental Disorders IV to ICD-10 criteria (with agreement between two psychiatrists). All participants were also assessed by either clinical genetic clinics or trained researchers for; dysmorphic features, co-morbid medical problems, and family history of ID and psychiatric disorders.

## Genetics analysis

Samples from Catalonia were analysed using the 400K Agilent platform (Agilent Technologies, Santa Clara, California, USA) at the Genetics Laboratory, UDIAT-Centre Diagnòstic, Parc Taulí Hospital Universitari. Samples from Leuven were analysed on the CytoSure ISCA oligoarray set (OGT, Oxford, UK) at the Constitutional Cytogenetics Unit of the Center of Human Genetics. Samples from England were analysed on the NimbleGen 135K platform (87%) (Roche NimbleGen, Madison, Wisconsin, USA) and the Cytoscan 750K platform (13%) (Affymetrics, Santa Clara, California, USA) at the North East Thames Regional Genetics Service Laboratory. In Catalonia, microarrays were processed according to manufacturer's specifications and Agilent Workbench 5.0, Feature Extraction and Cytogenomics software (Agilent Technologies, Santa Clara, California, USA) were used to render image analysis using the manufacturer's recommended settings. They were called CNVs when there were at least five consecutive probes with a minimum log2 ratio of 0.25. In Leuven, the Oxford Gene Technology (OGT) CytoSure ISCA oligoarray set (OGT, Oxford, UK) was processed containing 180k DNA oligonucleotides with a minimum resolution of 200 kb. Microarray hybridization and copy number variant (CNV) analysis were performed according the manufacturer's instructions. In England, microarrays were processed and CNVs were reported using clinical diagnostic laboratory protocols, in keeping with the Association for Clinical Genetic Science (ACGS) Best Practice Guidelines.

## References:

1. Glenn S, Cunningham C. Performance of young people with Down syndrome on the Leiter-R and British picture vocabulary scales*. J Intellect Disabil Res.* 2005; **49**: 239–44.

2. Vogels, A. *et al.* Presenting symptoms in adults with the 22q11 deletion syndrome. *Eur. J. Med. Genet.* **57,** 157–62 (2014).

3. Vanmarsenille, L. *et al.* Increased dosage of RAB39B affects neuronal development and could explain the cognitive impairment in male patients with distal Xq28 copy number gains. *Hum. Mutat.* **35,** 377–83 (2014).

4. Denayer, A. *et al.* Neuropsychopathology in 7 Patients with the 22q13 Deletion Syndrome: Presence of Bipolar Disorder and Progressive Loss of Skills. *Mol. Syndromol.* **3,** 14–20 (2012).

5. Hannes, F. D. *et al.* Recurrent reciprocal deletions and duplications of 16p13.11: the deletion is a risk factor for MR/MCA while the duplication may be a rare benign variant. *J. Med. Genet.* **46,** 223–232 (2009).

## Supplementary Table 1: Neurodevelopmental Risk CNV calling criteria

Hg19 positions and calling criteria, adopted from Kendall et al. (13)

| Locus Name | Critical Region | Calling Criteria |
| --- | --- | --- |
| 1p36 del | chr1:0-2500000 | Size >50% of critical region, affecting *GABRD* |
| 1p36 dup | chr1:0-2500000 | Size >50% of critical region, affecting *GABRD* |
| TAR del | chr1:145394955-145807817 | Size >50% of critical region |
| TAR dup | chr1:145394955-145807817 | Size >50% of critical region |
| 1q21.1 del | chr1:146527987-147394444 | Size >50% of critical region |
| 1q21.1 dup | chr1:146527987-147394444 | Size >50% of critical region |
| 1q24 del | chr1:169680333-173303337 | Size >50% of critical region |
| NRXN1 del | chr2:50145643-51259674 | Exonic deletions |
| 2p15-16.1 proximal dup | chr2:61245288-61414572 | Size >50% of critical region |
| 2q11.2 del | chr2:96742409-97677516 | Size >50% of critical region, affecting both *LMAN2L* and *ARID5A* |
| 2q13 del | chr2:111394040-112012649 | Size >50% of critical region |
| 2q13 dup | chr2:111394040-112012649 | Size >50% of critical region |
| 2q33.1 (*SATB2*) del | chr2:200134224-200325255 | Size >50% of critical region |
| 2q37 (*HDAC4*) del | chr2:239716679-243199373 | Size >50% of critical region, affecting *HDAC4* |
| 3p25.3 (*JAGN1* to *TATDN2*) dup | chr3:9932271-10322902 | Size >50% of critical region |
| 3p11.2 (*CHMP2B* to *POU1F1*) del | chr3:87267612-87531631 | Size >50% of critical region |
| 3q13 (*GAP43*) del | chr3:115332334-115504038 | Size >50% of critical region |
| 3q28-29 (*FGF12*) del | chr3:191859728-192126012 | Size >50% of critical region |
| 3q29 del | chr3:195720167-197354826 | Size >50% of critical region |
| Wolf-Hirschhorn del | chr4:1552030-2091303 | Size >50% of critical region |
| Wolf-Hirschhorn dup | chr4:1552030-2091303 | Size >50% of critical region |
| 4q21 (*BMP3*) del | chr4:81945477-81985327 | Size >50% of critical region |
| 5q14 (*MEF2C*) del | chr5:88011654-88200703 | Size >50% of critical region |
| Sotos syndrome del | chr5:175720924-177052594 | Size >50% of critical region |
| Williams-Beuren syndrome del | chr7:72744915-74142892 | Size >50% of critical region |
| WBS dup | chr7:72744915-74142892 | Size >50% of critical region |
| 8p23.1 del | chr8:8098990-11872558 | Size >26.5% of critical region (equal to min 1Mb affected) |
| 8p23.1 dup | chr8:8098990-11872558 | Size >26.5% of critical region (equal to min 1Mb affected) |
| 9p13 dup | chr9:32648800-38808255 | Size >50% of critical region |
| 9q34 dup | chr9:138460697-141036426 | Size >38.8% of critical region (equal to min 1Mb affected) |
| 10q11.21q11.23 dup | chr10:49390199-51058796 | Size >50% of critical region |
| 10q23 del | chr10:82045472-88931651 | Size > 14.5% of critical region (equal to min 1Mb affected), including *NRG3* and *GRID1* |
| Potocki-Shaffer syndrome del | chr11:43940000-46020000 | Size >50% of critical region, including *EXT2* |
| 12p13 dup | chr12:6471959-6825955 | Size >50% of critical region |
| PWS/AS del | chr15:22805313-28390339 | Size > 71.6% of critical region (equal to min 4Mb affected) |
| PWS/AS dup | chr15:22805313-28390339 | Size > 71.6% of critical region (equal to min 4Mb affected) |
| 15q11.2 BP1-BP2 del | chr15:22805313-23094530 | Size >50% of critical region |
| 15q13.3 del | chr15:31080645-32462776 | Size >50% of critical region |
| 15q24 del | chr15:72900171-78151253 | Size >50% of critical region |
| 15q24 dup | chr15:72900171-78151253 | Size >50% of critical region |
| 15q25 del | chr15:85139815-85716624 | Size >50% of critical region |
| 16p13.11 del | chr16:15511655-16293689 | Size >50% of critical region |
| 16p13.11 dup | chr16:15511655-16293689 | Size >50% of critical region |
| 16p12.1 del | chr16:21950135-22431889 | Size >50% of critical region |
| 16p11.2 distal del | chr16:28823196-29046783 | Size >50% of critical region |
| 16p11.2 distal dup | chr16:28823196-29046783 | Size >50% of critical region |
| 16p11.2 del | chr16:29650840-30200773 | Size >50% of critical region |
| 16p11.2 dup | chr16:29650840-30200773 | Size >50% of critical region |
| 17p13.3 del | chr17:1247834-2588909 | Exonic deletions; whole gene duplications |
| 17p13.3 dup | chr17:1247834-2588909 | Exonic deletions; whole gene duplications |
| Smith-Magenis syndrome del | chr17:16812771-20211017 | Size >50% of critical region |
| Potocki-Lupski syndrome dup | chr17:16812771-20211017 | Size >50% of critical region |
| 17q11.2 del | chr17:29107491-30265075 | Size >50% of critical region, affecting *NF1* |
| 17q11.2 dup | chr17:29107491-30265075 | Size >50% of critical region, affecting *NF1* |
| 17q12 del | chr17:34815904-36217432 | Size >50% of critical region |
| 17q12 dup | chr17:34815904-36217432 | Size >50% of critical region |
| 17q21.31 del | chr17:43705356-44164691 | Size >50% of critical region |
| 22q11.2 del | chr22:19037332-21466726 | Size >50% of critical region |
| 22q11.2 dup | chr22:19037332-21466726 | Size >50% of critical region |
| distal 22q11.2 del | chr22:21920127-23653646 | Size >50% of critical region |
| distal 22q11.2 dup | chr22:21920127-23653646 | Size >50% of critical region |
| Phelan-McDermid syndrome del | chr22:51113070-51171640 | Size >50% of critical region |
| Phelan-McDermid syndrome dup | chr22:51113070-51171640 | Size >50% of critical region |

##

## Supplementary Table 2: Reclassification of between site descrepancies

CNV regions which were reclassified due to discrepancies between sites, PubMed IDs (PMID) link to manuscripts describing pathogenicity.

| CNV region | Inital classification | Re-classification | Reason for re-classification |
| --- | --- | --- | --- |
| 2p16.3(50,882,091-50,949,412)x0 | VOUS likely pathogenic | VOUS likely benign | Non-exonic NRXN1 CNV - no literature proof of pathogenicity |
| 2p16.3(50,937,464-51,029,090)x0 | VOUS likely pathogenic | VOUS likely benign | Non-exonic NRXN1 CNV - no literature proof of pathogenicity |
| 15q11.2(22,698,579-23,249,693)x0 | VOUS likely pathogenic | Pathogenic | Literature evidence of pathogenicity. PMIDs: 21359847, 25689425 |
| 15q11.2(22,753,658-23,084,392)x0 | VOUS likely pathogenic | Pathogenic | Literature evidence of pathogenicity. PMIDs: 21359847, 25689425 |
| 15q11.2(22,753,658-23,187,967)x0 | VOUS likely pathogenic | Pathogenic | Literature evidence of pathogenicity. PMIDs: 21359847, 25689425 |
| 15q13.2q13.3(30,419,801-32,861,612)x0 | VOUS likely pathogenic | Pathogenic | Literature evidence of pathogenicity. PMIDs: 25077648, 26997942 |
| 16p11.2(29,652,360-30,199,696)x0 | VOUS likely pathogenic | Pathogenic | Literature evidence of pathogenicity. PMIDs: 19914906, 25064419 |
| 16p11.2(29,652,360-30,199,696)x3 | VOUS likely pathogenic | Pathogenic | Literature evidence of pathogenicity. PMIDs: 19914906, 26629640 |
| 16p13.12p12.3(14,622,055-17,409,257)x3 | VOUS likely pathogenic | Pathogenic | Literature evidence of pathogenicity. PMIDs: 21614007, 21150890 |

##

## Supplementary Table 3: Full descriptive summary of GENMID Cohort

Info from the three sites Catalonia, Leuven and England. Including summary of all psychiatric diagnoses found in ten or more individuals.

|  | GENMID | Catalonia | Leuven | England |
| --- | --- | --- | --- | --- |
| **Demographics** |  |  |  |  |
| N | 599 | 80 | 272 | 247 |
| Ratio (Male/Female) | 1.7 (376/223) | 0.9 (38/42) | 2.1 (184/88) | 1.7 (154/93) |
| Mean age (std.dev) | 43.2 (14.1) | 37.1 (9.8) | 46.2 (14.5) | 41.9 (14.1) |
| **ID Level** |  |  |  |  |
| Mild | 49.2% | 63.7% | 66.9% | 25.1% |
| Severe | 50.8% | 36.2% | 33.1% | 74.9% |
| **Psychiatric diagnoses** |  |  |  |  |
| Average number of co-morbid diagnoses (range) | 1.6 (1-5) | 1.8 (1-3) | 1.4 (1-4) | 1.7 (1-5) |
| F84 Pervasive developmental disorders | 148 (25%) | 9% | 22% | 32% |
| F29 Unspecified nonorganic psychosis | 121 (20%) | 12% | 30% | 12% |
| F61 Mixed and other personality disorders | 108 (18%) | 0% | 36% | 4% |
| Challenging behaviours | 95 (16%) | 62% | 1% | 17% |
| F32 Depressive episode | 86 (14%) | 4% | 3% | 30% |
| F41 Other anxiety disorders | 60 (10%) | 6% | 1% | 21% |
| F20 Schizophrenia | 49 (8%) | 4% | 8% | 9% |
| F31 Bipolar affective disorder | 47 (8%) | 2% | 7% | 11% |
| F90 Hyperkinetic disorders | 41 (7%) | 6% | 4% | 10% |
| F42 Obsessive-compulsive disorder | 37 (6%) | 16% | 1% | 9% |
| F43 Reaction to severe stress and adjustment disorders | 27 (5%) | 19% | 4% | 0% |
| F39 Unspecified mood disorder | 25 (4%) | 0% | 9% | 0% |

## Supplementary table 4: Likely pathogenic CNVs

Genetic and phenotypic data for participants with recurrent likely pathogenic CNVs. del = deletion, dup = duplication.

| **loci** | **chr** | **start** | **stop** | **size [Mb]** | **type** | **overlap group** | **Psychiatric Diagnosis (ICD-10)** | **ID Level** |
| --- | --- | --- | --- | --- | --- | --- | --- | --- |
| 1q21.1 | 1 | 143709609 | 143972412 | 0.263 | del | 1 | F31 Bipolar affective disorder, F84 Pervasive developmental disorders | severe |
| 1q21.1 | 1 | 143709609 | 143972412 | 0.263 | del | 1 | F29 Unspecified nonorganic psychosis, F31 Bipolar affective disorder | severe |
| 1q21.1 | 1 | 143902282 | 143972412 | 0.070 | del | 1 | F29 Unspecified nonorganic psychosis, F61 Mixed and other personality disorders | severe |
| 1q21.2 | 1 | 147471984 | 148773178 | 1.301 | del | 2 | F32 Depressive episode, F41 Other anxiety disorders, F90 Hyperkinetic disorders | severe |
| 1q21.2 | 1 | 147727771 | 147825519 | 0.098 | del | 2 | F43 Reaction to severe stress and adjustment disorders, F84 Pervasive developmental disorders, F90 Hyperkinetic disorders | mild |
| 1q32.1 | 1 | 206485595 | 206555480 | 0.070 | dup | 3 | Challenging behaviours, STEREotypies, F42 Obsessive-compulsive disorder | severe |
| 1q32.1 | 1 | 206485595 | 206555480 | 0.070 | del | 3 | F29 Unspecified nonorganic psychosis, F32 Depressive episode | severe |
| 3p26.3 | 3 | 606100 | 1330109 | 0.724 | dup | 4 | Challenging behaviours | mild |
| 3p26.3 | 3 | 1313271 | 1571571 | 0.258 | dup | 4 | F20 Schizophrenia, F61 Mixed and other personality disorders | mild |
| 5q22.2 | 5 | 111619275 | 111732130 | 0.113 | del | 5 | F29 Unspecified nonorganic psychosis | mild |
| 5q22.2 | 5 | 111666656 | 111718078 | 0.051 | del | 5 | F20 Schizophrenia, F42 Obsessive-compulsive disorder | mild |
| 7q31.1 | 7 | 111112186 | 111255558 | 0.143 | del | 6 | Challenging behaviours, F42 Obsessive-compulsive disorder, F84 Pervasive developmental disorders | severe |
| 7q31.1 | 7 | 111198987 | 111280493 | 0.082 | del | 6 | F43 Reaction to severe stress and adjustment disorders | severe |
| 9q21.32q21.33 | 9 | 86763706 | 87406685 | 0.643 | dup | 7 | F31 Bipolar affective disorder | severe |
| 9q21.32q21.33 | 9 | 86763706 | 87406685 | 0.643 | dup | 7 | F29 Unspecified nonorganic psychosis | mild |
| 14q11.2 | 14 | 21567757 | 21856165 | 0.288 | dup | 8 | F20 Schizophrenia | severe |
| 14q11.2 | 14 | 21740360 | 21963670 | 0.223 | del | 8 | F31 Bipolar affective disorder | severe |
| 14q11.2 | 14 | 21819565 | 22003106 | 0.184 | dup | 8 | F84 Pervasive developmental disorders | severe |
| 15q26.1 | 15 | 93357856 | 93536293 | 0.178 | del | 9 | F25 Schizoaffective disorder, F32 Depressive episode | severe |
| 15q26.1 | 15 | 93521186 | 93536293 | 0.015 | dup | 9 | Challenging behaviours, F31 Bipolar affective disorder | mild |
| 16p13.2 | 16 | 8911412 | 8942774 | 0.031 | dup | 10 | F61 Mixed and other personality disorders | mild |
| 16p13.2 | 16 | 8925643 | 9274514 | 0.349 | dup | 10 | F20 Schizophrenia, F61 Mixed and other personality disorders | mild |
| 17p13.1 | 17 | 6945270 | 7477779 | 0.533 | dup | 11 | F84 Pervasive developmental disorders | severe |
| 17p13.1 | 17 | 7394349 | 7890163 | 0.496 | dup | 11 | F61 Mixed and other personality disorders | mild |
| 18q23 | 18 | 77444688 | 78012819 | 0.568 | dup | 12 | F84 Pervasive developmental disorders | severe |
| 18q23 | 18 | 77466468 | 77887649 | 0.421 | dup | 12 | F32 Depressive episode, F61 Mixed and other personality disorders | mild |
| 20q11.21 | 20 | 31057960 | 49418758 | 18.361 | del | 13 | F84 Pervasive developmental disorders, F32 Depressive episode | severe |
| 20q11.23 | 20 | 35192298 | 35208091 | 0.016 | del | 13 | F84 Pervasive developmental disorders | mild |
| 20q13.12 | 20 | 43275709 | 43278342 | 0.003 | del | 14 | F61 Mixed and other personality disorders | severe |
| Xp22.33 | 23 | 60701 | 506573 | 0.446 | del | 15 | F84 Pervasive developmental disorders | severe |
| Xp22.33 | 23 | 296510 | 421339 | 0.125 | dup | 15 | Challenging behaviours | severe |
| Xq28 | 23 | 153030700 | 153104476 | 0.074 | dup | 16 | F23 Acute and transient psychotic disorders, F41 Other anxiety disorders, F42 Obsessive-compulsive disorder | severe |
| Xq28 | 23 | 153064834 | 153165618 | 0.101 | del | 16 | F31 Bipolar affective disorder | severe |
| Xq28 | 23 | 153127807 | 153154891 | 0.027 | dup | 16 | F32 Depressive episode, F84 Pervasive developmental disorders | mild |

##

## Supplementary table 5: Carriers of neurodevelopmental risk CNVs

Participants with CNVs at neurodevelopmental risk loci as described in Rees et al. 2016. N Expected, number of carriers we would expect based on ID/ASD frequencies (Rees et al. 2016) of the loci and our sample size of 599 participants, Rate(%) in GENMID and ID/ASD samples, ID level, other identified pathogenic CNVs and psychiatric diagnosis of the GENMID carriers.

| Locus | N Observed | N Expected | GENMID rate(%) | ID/ASD rate(%) | ID Level | Other pathogenic CNVs | Diagnosis |
| --- | --- | --- | --- | --- | --- | --- | --- |
| 22q11.2 del | 7 | 3.8 | 1.2 | 0.629 | Severe (4), Mild (3) | 2p16.3 del | F29 Unspecified nonorganic psychosis (3), F61 Mixed and other personality disorders (3), F31 Bipolar affective disorder (2), F39 Unspecified mood disorder (1) |
| 15q11.2 PWS/AS dup | 6 | 0.7 | 1.0 | 0.122 | Mild (5), Severe (1) |  | F84 Pervasive developmental disorders (3), Challenging behaviours (2), F29 Unspecified nonorganic psychosis (1), F31 Bipolar affective disorder (1), F39 Unspecified mood disorder (1), F40 Phobic anxiety disorders (1), F61 Mixed and other personality disorders (1), F91 Conduct disorder (1) |
| 15q13.3 del | 5 | 1.3 | 0.8 | 0.218 | Mild (4), Severe (1) |  | F32 Depressive episode (2), F41 Other anxiety disorders (2), Challenging behaviours (1), F29 Unspecified nonorganic psychosis (1), F31 Bipolar affective disorder (1), F84 Pervasive developmental disorders (1), F94 Disorders of social functioning with onset specific to childhood and adolescence (1) |
| 16p11.2 dup | 5 | 1.4 | 0.8 | 0.236 | Severe (5) |  | F84 Pervasive developmental disorders (3), F32 Depressive episode (2), F25 Schizoaffective disorder (1), F29 Unspecified nonorganic psychosis (1), F42 Obsessive-compulsive disorder (1), F90 Hyperkinetic disorders (1) |
| 2p16.3 NRXN1 del | 4 | 0.6 | 0.7 | 0.103 | Mild (2), Severe (2) | 22q11.21 del | F31 Bipolar affective disorder (2), Challenging behaviours (1), F22 Delusional disorder (1), F29 Unspecified nonorganic psychosis (1), F61 Mixed and other personality disorders (1) |
| 15q11.2 BP1-BP2 del | 4 | 3.7 | 0.7 | 0.625 | Mild (2), Severe (2) | Xp22.31 del | F29 Unspecified nonorganic psychosis (1), F31 Bipolar affective disorder (1), F41 Other anxiety disorders (1), F61 Mixed and other personality disorders (1) |
| 16p12.1 del | 4 | 0.7 | 0.7 | 0.124 | Mild (3), Severe (1) |  | F20 Schizophrenia (1), F23 Acute and transient psychotic disorders (1), F29 Unspecified nonorganic psychosis (1), F43 Reaction to severe stress and adjustment disorders (1), F61 Mixed and other personality disorders (1), F90 Hyperkinetic disorders (1) |
| 22q13.33 Phelan-McDermid del | 3 | 0.9 | 0.5 | 0.148 | Severe (3) |  | F84 Pervasive developmental disorders (2), Challenging behaviours (1), F29 Unspecified nonorganic psychosis (1), F31 Bipolar affective disorder (1) |
| 1q21.1 TAR dup | 2 | 0.9 | 0.3 | 0.155 | Mild (1), Severe (1) |  | F20 Schizophrenia (1), F43 Reaction to severe stress and adjustment disorders (1), F84 Pervasive developmental disorders (1), F90 Hyperkinetic disorders (1) |
| 4p16.3 Wolf-Hirschhorn dup | 2 | 0.2 | 0.3 | 0.038 | Mild (1), Severe (1) | 12p13.33p13.32 del | F29 Unspecified nonorganic psychosis (1), F84 Pervasive developmental disorders (1) |
| 7q11.23 WBS dup | 2 | 0.5 | 0.3 | 0.076 | Mild (2) |  | F39 Unspecified mood disorder (1), F61 Mixed and other personality disorders (1), F90 Hyperkinetic disorders (1) |
| 16p13.11 dup | 2 | 1.6 | 0.3 | 0.275 | Mild (1), Severe (1) |  | Challenging behaviours (1), F29 Unspecified nonorganic psychosis (1) |
| 16p11.2 distal del | 2 | 0.6 | 0.3 | 0.094 | Mild (1), Severe (1) |  | F23 Acute and transient psychotic disorders (1), F29 Unspecified nonorganic psychosis (1) |
| 16p11.2 del | 2 | 2.1 | 0.3 | 0.347 | Severe (2) |  | F29 Unspecified nonorganic psychosis (1), F41 Other anxiety disorders (1) |
| 22q11.2 dup | 2 | 1.6 | 0.3 | 0.260 | Mild (1), Severe (1) |  | F20 Schizophrenia (1), F29 Unspecified nonorganic psychosis (1) |
| 1q21.1 del | 1 | 1.2 | 0.2 | 0.201 | Mild (1) |  | F32 Depressive episode (1), F41 Other anxiety disorders (1) |
| 2q13 del | 1 | 0.3 | 0.2 | 0.057 | Mild (1) |  | F84 Pervasive developmental disorders (1) |
| 8p23.1 dup | 1 | 0.1 | 0.2 | 0.017 | Severe (1) |  | F39 Unspecified mood disorder (1), F61 Mixed and other personality disorders (1) |
| 15q11.2 PWS/AS del | 1 | 1.2 | 0.2 | 0.197 | Severe (1) |  | F41 Other anxiety disorders (1) |
| 16p13.11 del | 1 | 0.9 | 0.2 | 0.142 | Mild (1) |  | F61 Mixed and other personality disorders (1) |
| 16p11.2 distal dup | 1 | 0.5 | 0.2 | 0.083 | Mild (1) |  | Challenging behaviours (1) |
| 17p11.2 Potocki-Lupski dup | 1 | 0.3 | 0.2 | 0.055 | Mild (1) |  | F61 Mixed and other personality disorders (1) |
| 17q11.2 NF1 del | 1 | 0.2 | 0.2 | 0.039 | Severe (1) |  | Challenging behaviours (1), F84 Pervasive developmental disorders (1) |
| Total | 60 | 38.8 | 10.0 | 6.512 |  |  |  |

## Supplementary table 6: Clinically pathogenic CNVs not described in the literature associated CNVs

CNVs identified as pathogenic by clinical genetics services not described as ID associated loci in Rees et al 2016.

| Id | Position | Size [Mb] | Psychiatric Diagnosis (ICD-10) | ID Level |
| --- | --- | --- | --- | --- |
| ID 1 | 3q29(195,740,402-197,322,923)x3 | 1.6 | F29 Unspecified nonorganic psychosis, F61 Mixed and other personality disorders | mild |
| ID 2 | 4q34.3q35.2(182,074,317-190,807,379)x0 | 8.7 | F31 Bipolar affective disorder | severe |
| ID 3 | 5q14.3(90,098,388-98,399,833)x1 | 8.3 | F32 Depressive episode, F33 Recurrent depressive episodes, F41 Other anxiety disorders | mild |
| ID 4 | 6p25.3(195,429-7,392,549)x3 | 7.2 | F25 Schizoaffective disorder | mild |
| ID 5 | 9q21.11q21.13(71,031,677-76,816,695)x0 | 5.8 | F20 Schizophrenia | severe |
| ID 6 | 9q31.1q32(107056010-115867141)x1 | 8.8 | Challenging behaviours, F40 Phobic anxiety disorders, F43 Reaction to severe stress and adjustment disorders | severe |
| ID 7 | 10q26.12q26.3(122259702-135434178)x1 | 13.2 | Challenging behaviours, F42 Obsessive-compulsive disorder, F90 Hyperkinetic disorders | severe |
| ID 8 | 12p13.33p13.32(162,578-4,109,244)x0dn | 3.9 | F29 Unspecified nonorganic psychosis | severe |
| ID 9 | 12p13.1(13,754,549-13,762,809)x3 | 0.0 | Challenging behaviours | severe |
| ID 10 | 12p12.1(023432294-026233996)x1 | 2.8 | Challenging behaviours, F29 Unspecified nonorganic psychosis | mild |
| ID 11 | 12q21.2(79,534,629-84,535,827)x1 | 5.0 | F20 Schizophrenia | mild |
| ID 12 | 13q32.3(100,465,759-109,578,071)x3 | 9.1 | F84 Pervasive developmental disorders, F90 Hyperkinetic disorders | mild |
| ID 13 | 13q33.3q34(109,847,503-115,105,655)x1 | 5.3 | F61 Mixed and other personality disorders | mild |
| ID 14 | 15q11.2(24,826,468-25,342,534)x0 | 0.5 | F61 Mixed and other personality disorders | mild |
| ID 15 | 15q12(26,587,699-29,576,869)x1 | 3.0 | Obsessive traits, F84 Pervasive developmental disorders, F90 Hyperkinetic disorders | severe |
| ID 7 | 15q26.3(099168589-102480888)x3 | 3.3 | Challenging behaviours, F42 Obsessive-compulsive disorder, F90 Hyperkinetic disorders | severe |
| ID 16 | 19q13.32(45,741,741-47,268,131)x1 | 1.5 | F31 Bipolar affective disorder | severe |
| ID 17 | Xp22.33(1-155,270,560)x3 | 155.3 | F32 Depressive episode | mild |
| ID 18 | Xp22.33p11.22(169901-51101339)x3 | 50.9 | Challenging behaviours | severe |
| ID 19 | Xp22.31(6,451,638-8,139,295)x0 | 1.7 | F41 Other anxiety disorders | mild |
| ID 20 | Xp21.3p11.4(025816432-038085678)x2 | 12.3 | Damage and dysfunction, Challenging behaviours, F07 Personality and behavioural disorders due to brain disease, Damage and dysfunction | mild |
| ID 21 | Xq22.1(chrX:100,715,263-106,935,836)x1 | 6.2 | Challenging behaviours | severe |
| ID 22 | Xq24(118,883,247-123,283,287)x3 | 4.4 | Challenging behaviours | severe |
| ID 18 | Xq25q28(124642297-155227312)x1 | 30.6 | Challenging behaviours | severe |
| ID 17 | Y(1-59,373,566)x3 | 59.4 | F32 Depressive episode | mild |

## Supplementary figures


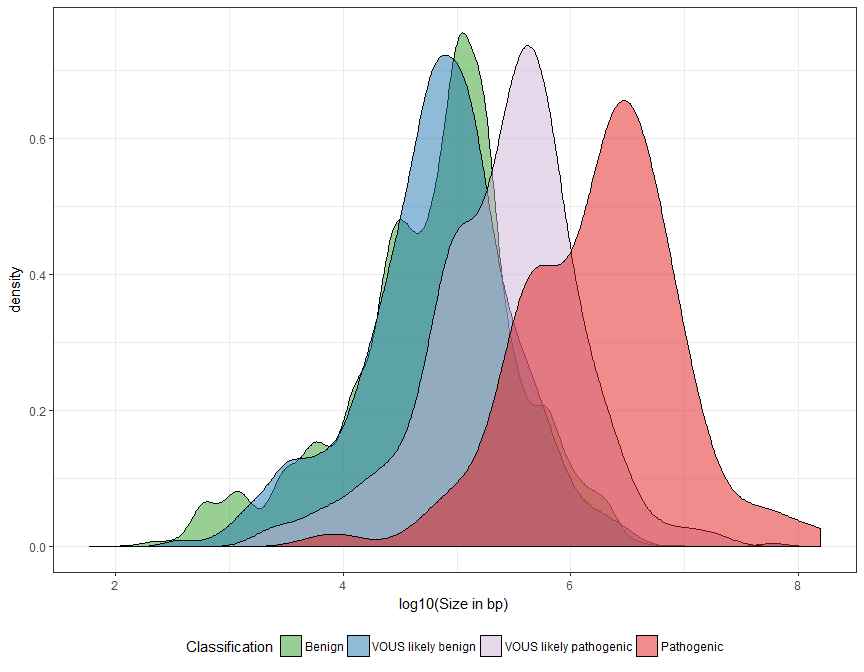


**Supplementary figure 1: Size distribution of GENMID CNVs by classification of pathogenicity.**

VOUS = Variant of unknown significance. The table below show size difference between pathogenic and other classified CNVs. Welch two sample t-test was performed to compare difference in mean between groups.

| Group A | Group B | Mean A (Mb) | Mean B (Mb) | Difference (95% CI) | p-value |
| --- | --- | --- | --- | --- | --- |
| Pathogenic | VOUS likely pathogenic | 6.36 | 0.71 | 5.65(1.6-9.7) | 0.0073 |
| Pathogenic | VOUS likely benign | 6.36 | 0.28 | 6.08(2-10) | 0.0039 |
| Pathogenic | Benign | 6.36 | 0.19 | 6.17(2.1-10) | 0.0035 |
| VOUS likely pathogenic | VOUS likely benign | 0.71 | 0.28 | 0.43(0.1-0.78) | 0.011 |
| VOUS likely pathogenic | Benign | 0.71 | 0.19 | 0.52(0.25-0.8) | 0.00024 |
| VOUS likely benign | Benign | 0.28 | 0.19 | 0.09(0.11-0.28) | 0.4 |


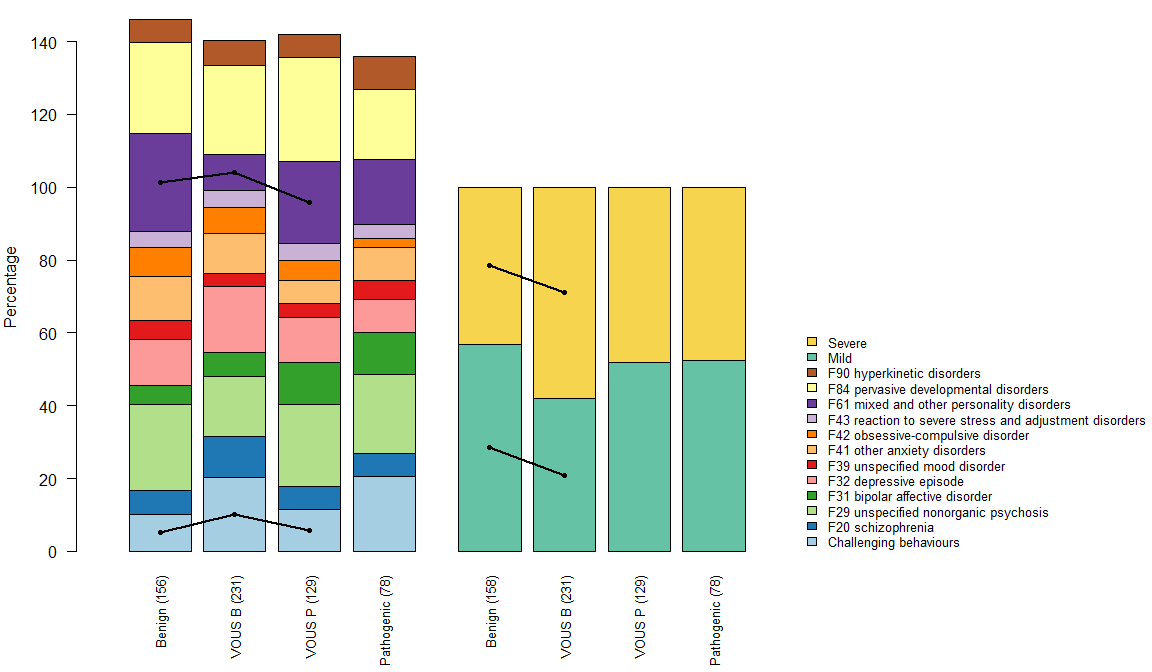


## Supplementary Figure 2: Association between CNV pathogenicity, diagnostic groups and ID severity

Bars 1-4 show the diagnostic rates of all diagnoses with 10 or more affected, for carriers with benign, variant of unknown significance likely benign (VOUS B), VOUS likely pathogenic (VOUS P) and pathogenic CNVs, as defined by the diagnostic services. Bars 5-8 show the frequency of mild and severe ID for individuals with benign, VOUS B, VOUS P and pathogenic CNVs. Lines indicate nominal statistical difference between groups P-value < 0.05 only adjacent groups within bar 1-4 and 5-8 have been tested.

Results for all group differences that showed a nominal statistically significant difference (p<0.05), 84 pairwise comparisons were done. Based on comparisons between diagnostic rates of all diagnoses with 10 or more affected and ID severity, for carriers with benign, Variant of unknown significance likely benign (VOUS B), VOUS likely pathogenic (VOUS P) and pathogenic CNVs.

| Group1 | Group2 | Diagnosis/ID-level | Estimate | p-value |
| --- | --- | --- | --- | --- |
| Benign | VOUS B | Challenging behaviours | 0.45 | 1.11e-02 |
| Benign | VOUS B | F61 mixed and other personality disorders | 3.32 | 2.35e-05 |
| VOUS B | VOUS P | Challenging behaviours | 1.94 | 4.14e-02 |
| VOUS B | VOUS P | F61 mixed and other personality disorders | 0.38 | 1.67e-03 |
| Benign | VOUS B | mild | 1.83 | 3.91e-03 |
| Benign | VOUS B | severe | 0.55 | 3.91e-03 |
